# Experimental Validation of a Model-Free High-Order Sliding Mode Controller with Finite-Time Convergence for Trajectory Tracking of Autonomous Underwater Vehicles

**DOI:** 10.3390/s22020488

**Published:** 2022-01-09

**Authors:** Josué González-García, Alfonso Gómez-Espinosa, Luis Govinda García-Valdovinos, Tomás Salgado-Jiménez, Enrique Cuan-Urquizo, Jesús Arturo Escobedo Cabello

**Affiliations:** 1 Tecnologico de Monterrey, Escuela de Ingenieria y Ciencias, Av. Epigmenio González 500, Fracc. San Pablo, Queretaro 76130, Mexico; a01208772@itesm.mx (J.G.-G.); ecuanurqui@tec.mx (E.C.-U.); arturo.escobedo@tec.mx (J.A.E.C.); 2 Center for Engineering and Industrial Development-CIDESI, Energy Division, Queretaro 76125, Mexico; tsalgado@cidesi.edu.mx

**Keywords:** AUV, SMC, finite-time, trajectory tracking

## Abstract

Several control strategies have been proposed for the trajectory tracking problem of Autonomous Underwater Vehicles (AUV). Most of them are model-based, hence, detailed knowledge of the parameters of the robot is needed. Few works consider a finite-time convergence in their controllers, which offers strong robustness and fast convergence compared with asymptotic or exponential solutions. Those finite-time controllers do not permit the users to predefine the convergence time, which can be useful for a more efficient use of the robot’s energy. This paper presents the experimental validation of a model-free high-order Sliding Mode Controller (SMC) with finite-time convergence in a predefined time. The convergence time is introduced by the simple change of a time-base parameter. The aim is to validate the controller so it can be implemented for cooperative missions where the communication is limited or null. Results showed that the proposed controller can drive the robot to the desired depth and heading trajectories in the predefined time for all the cases, reducing the error by up to 75% and 41% when compared with a PID and the same SMC with asymptotic convergence. The energy consumption was reduced 35% and 50% when compared with those same controllers.

## 1. Introduction

Autonomous Underwater Vehicles (AUV) have permitted us to deepen the knowledge of the oceans and seafloors. The use of AUVs in tasks such as structural inspection [1,2], environmental risks detection [3], and mapping underwater structures [4], among others, increases the safety of the mission and the reliability of their results [5], and reduces the operational costs considerably [6]. However, due the challenging conditions in the underwater environment, a high precision autonomous navigation is difficult to achieve [7]. Autonomous navigation can be performed by different methods such as waypoint tracking, path following, and trajectory tracking [8]. In the waypoint tracking method, the vehicle navigates throughout a set of pre-defined waypoints. This is the easier method to be implemented, however, it could result in some uncertainty in the trajectory followed by the vehicle between two waypoints [9]. The path following navigation ensures that the vehicle follows a desired path to reach its destination, which is geometrically defined in Cartesian coordinates [10]. In the trajectory tracking problem, the vehicle must follow a time-parametrized path, meaning it must go to a certain point in a given time [11]. The integration of time and space restrictions makes trajectory tracking the most complex of the referred autonomous navigation methods. Considering this, the highly non-linear dynamics of the AUV and the presence of external disturbances demands the development of new control methods to fulfill the extremely challenging problem of trajectory tracking in AUVs.

Proportional Integrate Derivative (PID) control was extensively used at the early stages of AUV tracking control [12,13,14]. PID controllers have a simple structure that allows an easier implementation. They are tuned to deal with very specific conditions and any change on them will cause a performance drop. Additionally, they do not consider non-linearities, which are highly present in the AUVs dynamics and the underwater environment. An alternative to deal with traditional controllers’ issues is to include an intelligent algorithm [15,16] in the control law to achieve an adjustment of the controller parameters or self-tuning. This is known as adaptive control, which does not require an exact model of the vehicle and only a small amount of prior knowledge suffices. Backstepping control [17] and Sliding Mode Control (SMC) [18] are other common control methods widely used in the trajectory tracking of AUVs. The control methods used for AUVs trajectory tracking discussed before have an asymptotic or exponential convergence to the trajectory. A finite-time convergence controller would have many advantages such as faster convergence time, higher accuracy, and better anti-disturbance capability [19].

### Related Work

Limited literature can be found related to control methods with finite-time convergence for AUV trajectory tracking. Ramezani-al et al. [20] proposed an SMC with an adaptive gain to eliminate the effects of external chattering and noise vulnerability. In numerical simulations, the proposed controller drives the vehicle to the desired trajectory in a limited time. Yu et al. [21] achieved finite-time convergence trajectory tracking of AUVs in presence of model parameter perturbation and ocean currents. The controller is based on a PID-SMC combination that globally stabilizes all tracking errors in a finite-time. Numerical simulations showed absolute tracking errors up to 20 cm in the absence of external disturbances. Qiao et al. [22] used two adaptive integral terminal SMC schemes to achieve trajectory tracking on AUVs. This controller yields finite-time convergence while dealing with dynamic uncertainties and external disturbances. The performance of the proposal was tested by numerical simulations resulting in robustness of 2% in position tracking. In those works, the controlled trajectory converges to the reference in a time that depends either on the vehicle parameters, its initial position, or the own controller parameters and gains. Therefore, prior knowledge of the vehicle is required to estimate the convergence time, and a different initial position and changes on the controller parameters will affect it. González-García et al. [23] presented a model-free high-order SMC with finite-time convergence aiming for cooperative AUVs missions. On contrary to previous works, the finite-time for the convergence can be predefined by the user through the time-base parameter $t_{b}$, the value of which—in seconds—is only limited by the thruster’s capabilities. The arbitrary setting of the time-base parameter does not affect the robustness and performance of the controller once it meets the desired trajectory and can be used to demand a faster or slower response from the vehicle depending on what the mission requires. Numerical simulations were performed and results showed an outstanding performance when compared with traditional controllers. Even in the presence of strong ocean currents, the error was zero in the predefined time in all the cases simulated.

All of the works discussed before were limited to numerical simulations. However, due to the limitations present in real experiments, such as sensor resolution, measurement rate, processor speed, and so, it is quite possible that a controller does not perform as expected when programmed in a real system. The performance of a model-free high-order SMC with finite-time convergence in a predefined time is validated by real experiments in this paper. Being able to arbitrarily select the convergence time for the controller opens the options for better management of the vehicle energy consumption. It would also be an advantage to coordinate a group of AUVs in cooperative tasks. The performance of the controller was compared with a PID and an asymptotic 2nd-order SMC. The experiments were performed using the BlueROV2 robot which was modified to be able to follow predefined trajectories autonomously. The experiments consisted of a depth and heading control to follow a predefined sinusoidal trajectory and were carried out in a pool with 1 m depth.

The rest of the document is structured as: Section 2 describes the generalities of underwater vehicles, the control algorithm to be validated, and the BlueROV2 vehicle configuration. Section 3 describes the experimental set up including the BlueROV2 modifications and controller’s laws and parameters. Section 4 shows the results of the experiments performed and their discussion, and Section 5 contains the concluding remarks.

## 2. Materials and Methods

General equations for kinematics and hydrodynamics of the AUVs are introduced in this section along with the particularities of the robot used in the experiments. After that, the model-free high-order SMC with finite-time convergence in predefined time is also introduced.

### 2.1. Autonomous Underwater Vehicles Kinematics and Hydrodynamics

**Figure 1:**
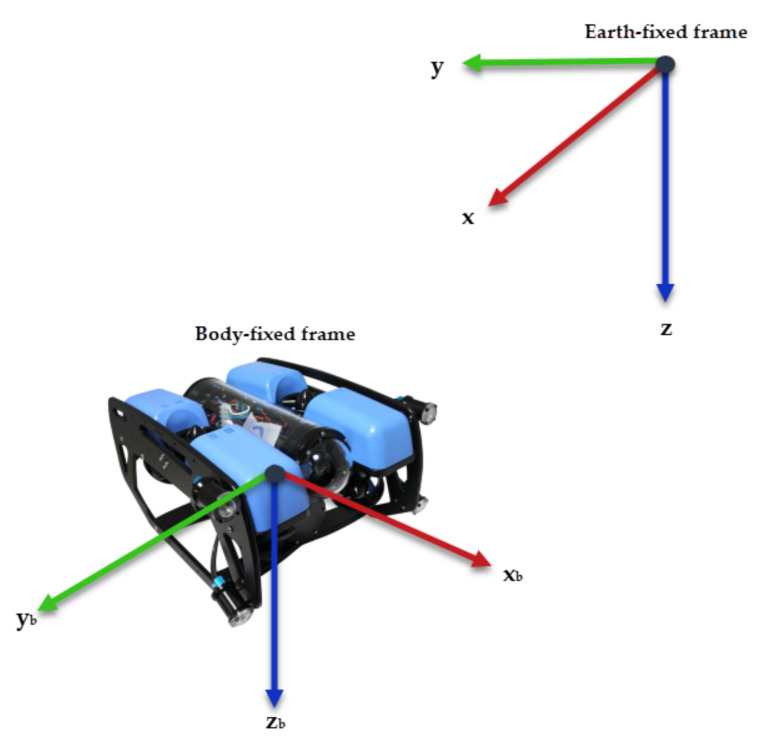
Reference frames for an AUV.

Fossen [24] describes the kinematics of underwater vehicles using two reference frames: an Earth-fixed frame and a Body-fixed frame. As shown in Figure 1, the orthonormal axes are denoted as *x*, *y*, and *z* for the Earth-fixed frame and $x_{b}$, $y_{b}$, and $z_{b}$ for the Body-fixed frame.

There is a convention for the notation of underwater vehicle’s position, orientation, and velocities accepted by the Society of Naval Architects and Marine Engineers (SNAME) [24], which also defines the forces and moments applied to the vehicle. Considering this nomenclature, the vehicle’s position regarding the Earth-fixed frame (η) and its velocities regarding the Body-fixed frame (ν) can be described as

(1) $$\eta ={\left(x,y,z,\phi ,\theta ,\psi \right)}^{T},$$

(2) $$\nu ={\left(u,v,w,p,q,r\right)}^{T},$$

and the forces and moments with respect to the Body-fixed frame (τ) as

(3) $$\tau ={\left(X,Y,Z,K,M,N\right)}^{T}.$$

The hydrodynamical model for underwater vehicles, as described by Newton–Euler equations [24], is given by

(4) $$M\dot{\nu }+C\left(\nu \right)\nu +D\left(\nu \right)\nu +g\left(\eta \right)=\tau +\omega ,$$

(5) $$\tau =B_{t}u_{t},$$

where

$M\epsilon R^{6\times 6}$ is the inertial and added mass matrix,
$C\epsilon R^{6\times 6}$ is the rigid body and added mass centripetal and Coriolis matrix,
$D\epsilon R^{6\times 6}$ is the hydrodynamic damping matrix,
$g\epsilon R^{6\times 1}$ is the restitution forces vector,
$\tau \epsilon R^{6\times 1}$ is the control signal vector,
$B_{t}\epsilon R^{6\times 6}$ is the thruster allocation matrix,
$u_{t}\epsilon R^{6\times 1}$ is a vector containing the force generated by the thrusters, and
$\omega \epsilon R^{6\times 6}$ represents environmental disturbances.

According to Fossen [24], the model given by Equation (4) can be expressed in the Earth-fixed frame applying kinematic transformations as follows

(6) $$\dot{\eta }=J\left(\eta_{2}\right)\nu ⟷\nu =J^{-1}\left(\eta_{2}\right)\dot{\eta },$$

(7) $$\ddot{\eta }=J\left(\eta_{2}\right)\dot{\nu }+\dot{J}\left(\eta_{2}\right)\nu ⟷\dot{\nu }=J^{-1}\left(\eta_{2}\right)\left[\ddot{\eta }-J\left(\eta_{2}\right)\nu \right],$$

where

(8) $$J\left(\eta_{2}\right)=\left[\begin{array}{cc} J_{1}\left(\eta_{2}\right) & 0_{3\times 3}\\ 0_{3\times 3} & J_{2}\left(\eta_{2}\right) \end{array}\right],$$

with

(9) $$J_{1}\left(\eta_{2}\right)=\left[\begin{array}{ccc} c_{\psi }c_{\theta } & -s_{\psi }c_{\phi }+s_{\phi }s_{\theta }c_{\psi } & s_{\psi }s_{\phi }+s_{\theta }c_{\psi }c_{\phi }\\ s_{\psi }c_{\phi } & c_{\psi }c_{\phi }+s_{\phi }s_{\theta }s_{\psi } & -c_{\psi }s_{\phi }+s_{\theta }s_{\psi }c_{\phi }\\ -s_{\theta } & s_{\phi }c_{\theta } & c_{\phi }c\theta \end{array}\right],$$

(10) $$J_{2}\left(\eta_{2}\right)=\left[\begin{array}{ccc} 1 & s_{\phi }t_{\theta } & c_{\phi }t\theta \\ 0 & c_{\phi } & -s_{\phi }\\ 0 & s_{\phi }/c_{\theta } & c_{\phi }/c_{\theta } \end{array}\right],and$$

(11) $$\eta_{2}=\left[\phi ,\theta ,\psi \right],$$

$c_{angle}$, $s_{angle}$, and $t_{angle}$ are abbreviations for cos(angle), sin(angle), and tan(angle), respectively.

After some mathematical manipulation, the resulting expression for the hydrodynamical model in the Earth-fixed frame is

(12) $$M_{\eta }\left(\eta \right)\ddot{\eta }+C_{\eta }\left(\nu ,\eta \right)\dot{\eta }+D_{\eta }\left(\nu ,\eta \right)\dot{\eta }+g_{\eta }\left(\eta \right)=\tau_{\eta },$$

where

(13) $$\begin{array}{c} M_{\eta }\left(\eta \right)=J^{-T}\left(\eta \right)MJ^{-1}\left(\eta \right), \end{array}$$

(14) $$\begin{array}{c} C_{\eta }\left(\nu ,\eta \right)=J^{-T}\left(\eta \right)\left[C\left(\nu \right)-MJ^{-1}\left(\eta \right)\dot{J}\left(\eta \right)\right]J^{-1}\left(\eta \right), \end{array}$$

(15) $$\begin{array}{c} D_{\eta }\left(\nu ,\eta \right)=J^{-T}\left(\eta \right)D\left(\nu \right)J^{-1}\left(\eta \right), \end{array}$$

(16) $$\begin{array}{c} g_{\eta }\left(\eta \right)=J^{-T}\left(\eta \right)g\left(\eta \right), \end{array}$$

(17) $$\begin{array}{c} \tau_{\eta }\left(\eta \right)=J^{-T}\left(\eta \right)\tau . \end{array}$$

### 2.2. Finite-Time Controller with Convergence in a Predefined Time

The High-order SMC with finite-time convergence used in this work is based in the proposal by García-Valdovinos et al. [25,26,27] and was adapted for trajectory tracking of AUVs in [23] as follows:

Consider that Equation (12) is linearly parametrizable by the product of a regressor $Y\left(\eta ,\dot{\eta },\ddot{\eta }\right)\epsilon R^{n\times p}$, which is composed of known nonlinear functions and a vector $\theta \epsilon R^{p}$ with constant parameters. The parametrization of Equation (12) can be rewritten in terms of a nominal reference ${\dot{\eta }}_{r}$ and its derivative ${\ddot{\eta }}_{r}$ as

(18) $$M_{\eta }\left(\eta \right){\ddot{\eta }}_{r}+C_{\eta }\left(\nu ,\eta \right){\dot{\eta }}_{r}+D_{\eta }\left(\nu ,\eta \right)S_{r}=\tau_{\eta }-Y\left(\eta ,\dot{\eta },\ddot{\eta }\right)\theta .$$

Subtracting Equation (18) from both sides of Equation (12) leads to the open-loop error dynamics expression

(19) $$M_{\eta }\left(\eta \right){\dot{S}}_{r}+C_{\eta }\left(\nu ,\eta \right)S_{r}+D_{\eta }\left(\nu ,\eta \right)S_{r}=\tau_{\eta }-Y\left(\eta ,\dot{\eta },\ddot{\eta }\right)\theta ,$$

where $S_{r}$ is known as the extended error computed as

(20) $$S_{r}=\dot{\eta }-{\dot{\eta }}_{r},$$

considering the nominal reference

(21) $${\dot{\eta }}_{r}={\dot{\eta }}_{d}-\alpha \tilde{\eta }+S_{d}-K_{i}\int_{0}^{t}sign\left(S_{\eta }\right)d\sigma ,$$

where the tracking error of the position is given by $\tilde{\eta }=\eta -\eta_{d}$, $\eta_{d}$ is the desired position, α and $K_{i}$ are diagonal positive definite n×n gain matrices, sign(x) is the signum function of the *x* vector, and

(22) $$\begin{array}{c} S=\dot{\tilde{\eta }}+\alpha \tilde{\eta }, \end{array}$$

(23) $$\begin{array}{c} S_{d}=S\left(t_{0}\right)e^{-kt}, \end{array}$$

(24) $$\begin{array}{c} S_{\eta }=S-S_{d}, \end{array}$$

with k>0. The extended error can be now rewritten as

(25) $$S_{r}=S_{\eta }+K_{i}\int_{0}^{t}sign\left(S_{\eta }\right)d\sigma ,$$

resulting in the model-free second-order SMC which control law is defined as

(26) $$\tau_{\eta }=-K_{d}S_{r}.$$

**Figure 2:**
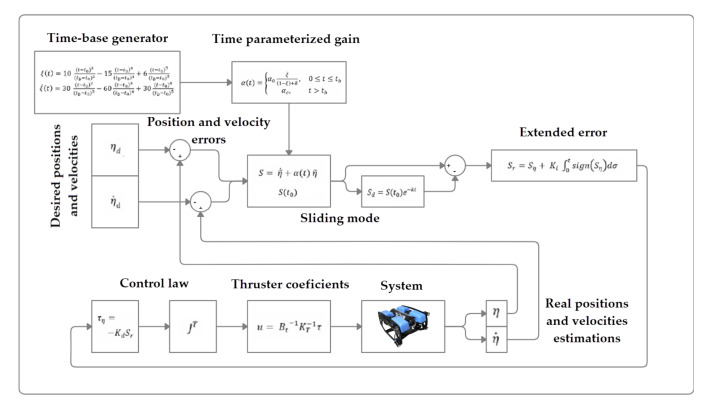
Model-free high-order SMC with finite time convergence block diagram.

A stability analysis of the controller just described can be found in [27]. To achieve finite-time convergence with this model-free high-order SMC, a Time-Base Generator (TBG) is used as described in [25,26]. This TBG is a scalar function providing a smooth 0 to 1 transition. The duration of that transition is controllable by a predefined time-base $t_{b}$. The derivative of this function provides a bell-shaped speed profile. The functions used for the TBG are

(27) $$\xi \left(t\right)=10\frac{{\left(t-t_{0}\right)}^{3}}{{\left(t_{b}-t_{0}\right)}^{3}}-15\frac{{\left(t-t_{0}\right)}^{4}}{{\left(t_{b}-t_{0}\right)}^{4}}+6\frac{{\left(t-t_{0}\right)}^{5}}{{\left(t_{b}-t_{0}\right)}^{5}}$$

and

(28) $$\dot{\xi }\left(t\right)=30\frac{{\left(t-t_{0}\right)}^{2}}{{\left(t_{b}-t_{0}\right)}^{3}}-60\frac{{\left(t-t_{0}\right)}^{3}}{{\left(t_{b}-t_{0}\right)}^{4}}+30\frac{{\left(t-t_{0}\right)}^{4}}{{\left(t_{b}-t_{0}\right)}^{5}},$$

where $t_{0}$ represents the initial time. Equations (27) and (28) are subjected to the following conditions: $\xi \left(t_{0}\right)=\dot{\xi }\left(t_{0}\right)=\dot{\xi }\left(t_{b}\right)=0$. Then, ξ(t) is used to parameterize the gain α in Equation (22) as time-varying as follows

(29) $$\alpha \left(t\right)=\left\{\begin{array}{cc} \alpha_{0}\frac{\dot{\xi }}{(1-\xi )+\delta } & 0\leq t\leq t_{b}\\ \alpha_{c} & t>t_{b} \end{array}\right.,$$

with $\alpha_{0}=1+\epsilon$, 0<ϵ≪1, 0<δ≪1, and $\alpha_{c}>0$. A diagram of the complete control scheme is showed in Figure 2.

**Remark** **1.** *The time-base parameter $t_{b}$ can be chosen arbitrarily and does not depends on the initial conditions or controller parameters. The convergence of the controlled trajectory will occur at the selected $t_{b}$, which means that a smaller value for $t_{b}$ will produce a faster convergence, and a bigger $t_{b}$ value will produce a slower convergence.*

### 2.3. BlueROV2 Robot

**Figure 3:**
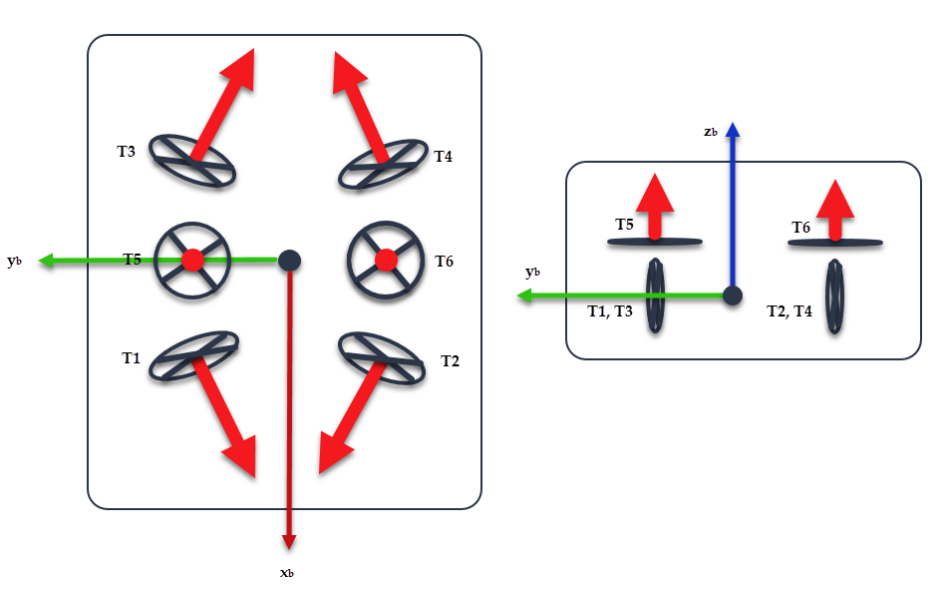
BlueROV2 thruster configuration. (**Left**) Top view. (**Right**) Front view.

The BlueROV2 from Blue Robotics^®^ is a Remotely Operated Vehicle (ROV) with a 6-Thruster vectored configuration as shown in Figure 3. It has an open-source software and hardware configuration, which make it suitable for research purposes.

A complete set of the BlueROV2 physic and hydrodynamic parameters is listed in [23]. However, this work considers a model-free control algorithm, which means that only the thruster allocation matrix $B_{t}$ is needed. From the BlueROV2 thruster distribution, $B_{t}$ is defined as

(30) $$B_{t}=\left[\begin{array}{cccccc} 0.7071 & 0.7071 & -0.7071 & -0.7071 & 0 & 0\\ -0.7071 & 0.7071 & -0.7071 & 0.7071 & 0 & 0\\ 0 & 0 & 0 & 0 & -1 & -1\\ 0 & 0 & 0 & 0 & 0.115 & -0.115\\ 0 & 0 & 0 & 0 & 0 & 0\\ -0.1773 & 0.1773 & -0.1773 & 0.1773 & 0 & 0 \end{array}\right].$$

### 2.4. Exact Differentiatior

The tracking error derivative is needed to compute the control signal. That means that the velocities of the robot need to be estimated. The easiest way to do that is through a simple Euler differentiatior. However, this differentiatior is quite sensitive to noise and will produce an inaccurate estimation of the robot velocities. An alternative is to use an exact differentiatior as the proposed by Davila et al. [28].

## 3. Experimental Set-Up

**Figure 4:**
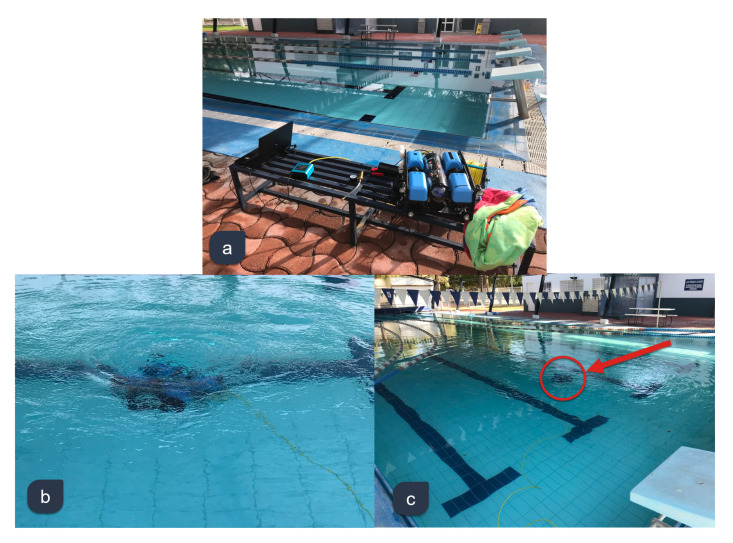
Experimental set-up at Tecnologico de Monterrey, Campus Querétaro. (**a**) Ground control station at a side of the semi-Olympic pool. (**b**) BlueROV2 deployed into the water. (**c**) Autonomous trajectory tracking mission.

The BlueROV2 was modified in its hardware and software to be able to autonomously follow predefined depth and yaw trajectories. The proposed model-free high-order SMC with finite-time convergence was tested in different experiments and its performance was compared with traditional PID and 2nd-order SMC. The experimental set up can be observed in the Figure 4.

The experiments were performed in the Tecnologico de Monterrey, Campus Queretaro semi-olympic swimming pool. All the runs were made in the same robot—BlueROV2—using three full-charged batteries—one for every control scheme—from 9:00 a.m. to 12:00 p.m. This with the aim of perform the experiments under the same conditions. No external disturbances were introduced to the experiments. An OMEN O15 Laptop with a Intel CORE i7 processor running UBUNTU 16 as operative system was used as ground control station.

### 3.1. Hardware

**Figure 5:**
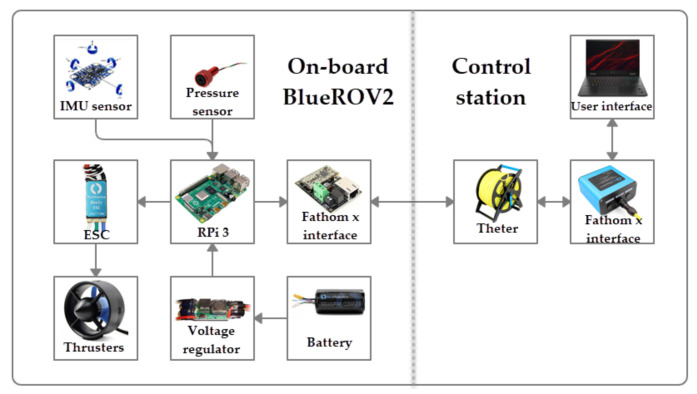
BlueROV2 hardware configuration.

Inside the BlueROV2, a Raspberry Pi^®^ 3 (RPi) acts as the processor, and it is in charge of running the control algorithms and managing the different sensors. The Rpi runs Lubuntu as operative system, and it is connected to a control station through a tether. A BAR-30 high-resolution pressure sensor from BlueRobotics^®^ is connected to the robot through an I${}^{2}$C port in the RPi to measure the depth *z* of the vehicle. A Smart sensor BNO-055 from Bosh^®^ is connected to a serial port of the Rpi and is used to estimate the orientations ϕ,θ,ψ. The thruster’s speeds are controlled by Pulse Wide Modulation (PWM) signals from the RPi, which goes through a set of 30 A Electronic Speed Controllers (ESC). Finally, a 14.8 V, 18A Ah battery supplies energy for the whole electronics. A diagram of the hardware configuration in the BlueROV2 used for the experiments is shown in Figure 5.

### 3.2. Software

**Figure 6:**
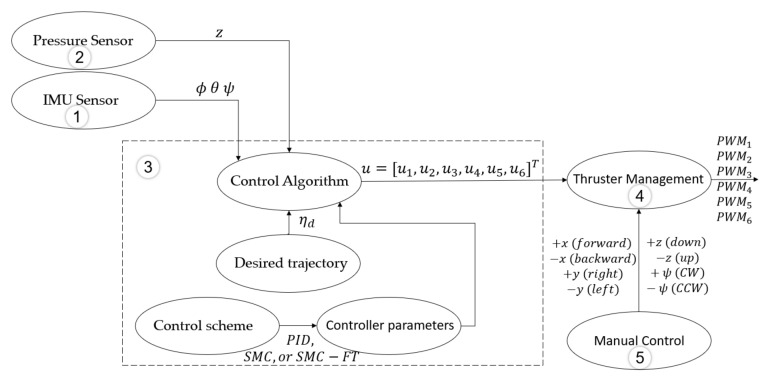
BlueROV2^®^ software configuration.

The Robot Operating System (ROS) in its Kinetic version was used to program the algorithms to manage the sensors, thruster’s speed, and control schemes. Five nodes were used in total (shown in Figure 6).

The *IMU sensor* node (1) manages the BNO-055 sensor and it is able to obtain a new set of ϕ,θ, and ψ orientations at a 100 Hz rate. The *Pressure Sensor* node (2) manages the Bar-30 sensor and obtains a new *z* position at a 10 Hz rate. Both nodes publish the estimated positions so they can be used as inputs to the *Control Algorithm* node (3). In this node, the different control algorithms were programmed along with a user interface to provide the user the option of selecting between the PID control, 2nd-Order SMC, and the model-free high-order SMC with finite-time convergence. The user can also modify the controller parameters and gains from this interface. In the algorithm, the desired position is generated as defined by the user throughout the user interface—either as a set point or as time-variant—and compared with the actual position estimated by the sensors. Then, the selected control law is applied and a thruster’s coefficients vector is computed and published as a result. The *Thruster Managment* node (4) reads the thruster’s coefficients vector and converts it to the proper PWM signal for each thruster to be generated by the RPi peripherals. The *Manual Control* node (5) is used prior to the experiment to position the BlueROV2 in its initial position.

### 3.3. Time-Parameterized Trajectories

A sinusoidal trajectory was chosen for the vehicle to track both depth ($z_{d}$), and yaw ($\psi_{d}$). Those trajectories are given by

(31) $$z_{d}=z_{i}+A_{z}sin\left(\omega t\right),\mathrm{and}\;\psi_{d}=\psi_{i}+A_{\psi }sin\left(\omega t\right),$$

where $z_{i}$ and $\psi_{i}$ are the initial desired position or offset, $A_{z}$ and $A_{\psi }$ are the desired amplitudes, and ω is the frequency for the signal. Then, the desired velocities $\dot{z_{d}}$ and $\dot{\psi_{d}}$ are

(32) $$\dot{z_{d}}=-\omega A_{z}cos\left(\omega t\right),\mathrm{and}\;\dot{\psi_{d}}=-\omega A_{\psi }cos\left(\omega t\right).$$

### 3.4. Control Algorithms

Experiments were carried out using three different control laws for the trajectory tracking of the robot. The aim was to compare the performance of the proposed algorithm with other common controllers used for this purpose.

#### 3.4.1. PID Control

The control law used for this controller is

(33) $$\tau_{\eta }=-k_{p}\tilde{\eta }-k_{i}\int \tilde{\eta }dt-k_{d}\dot{\tilde{\eta }},$$

where $k_{p}$, $k_{i}$, and $k_{d}$ are the proportional, integral, and derivative gain matrices, respectively. For the experiments on this work, the gain matrices that gave the best results are

$$k_{p}=diag\left[0,0,100,100,0,100\right],\;k_{i}=diag\left[0,0,1,1,0,1\right],\mathrm{and}\;$$

(34) $$k_{d}=diag\left[0,0,80,10,0,80\right].$$

#### 3.4.2. Model-Free High-Order SMC (Asymptotic)

The control law used for this controller is

(35) $$\tau_{\eta }=-K_{d}S_{r},$$

with

$$S_{r}=S_{\eta }+K_{i}\int_{0}^{t}sign\left(S_{\eta }\right)d\sigma ,\;S_{\eta }=S-S_{d},$$

(36) $$S_{d}=S\left(t_{0}\right)e^{-\kappa t},\mathrm{and}\;S=\dot{\tilde{\eta }}+\alpha \tilde{\eta }.$$

where α>0, κ>0, $K_{i}$, and $K_{d}$ are constant gains, $t_{0}$ is the initial time, and $sign(S_{\eta })$ is the sign function of $S_{\eta }$. For the experiments on this work, the gain set that gave the best results is

$$\alpha =10,\;\kappa =5,\;K_{i}=diag\left[0,0,0.05,0.005,0,0.001\right],\mathrm{and}\;$$

(37) $$K_{d}=diag\left[0,0,100,0.1,0,0.1\right].$$

#### 3.4.3. Model-Free High-Order SMC (Finite-Time)

The control law used for this controller is the same as the previous section (Equation (35)). To achieve finite-time convergence, the α gain in Equation (36) is substituted by the time-variant gain α(t) defined in Equation (29), with $\alpha_{c}=10$ for depth control, $\alpha_{c}=5$ for heading control, and, in both cases, $\alpha_{0}=1.005$ and δ=0.001. The rest of gain parameters (κ, $K_{i}$, and $K_{d}$) stays as defined in Equation (37).

### 3.5. Exact Differentiator Algorithm

The algorithm proposed for the exact differentiator is given by

$$\dot{j_{0}}=w_{0},$$

$$w_{0}=-\lambda_{1}{\left(j_{0}-f\left(t\right)\right)}^{1/2}\times sign\left(j_{0}-f\left(t\right)\right)+j_{1},and$$

(38) $$\dot{j_{1}}=-\lambda_{2}\times sign\left(j_{0}-f\left(t\right)\right),$$

where $\lambda_{1}=1.5$ and $\lambda_{2}=1.1$ are constant, f(t) is the signal we want to differentiate, and sign() stands for the sign function. After a brief adjustment time, the following is considered as true

(39) $$j_{0}=f\left(t\right),\;j_{1}=\dot{f}\left(t\right),$$

$j_{0}=0$ and $j_{1}=0$ were considered as initial values for the algorithm.

## 4. Results and Discussion

**Figure 7:**
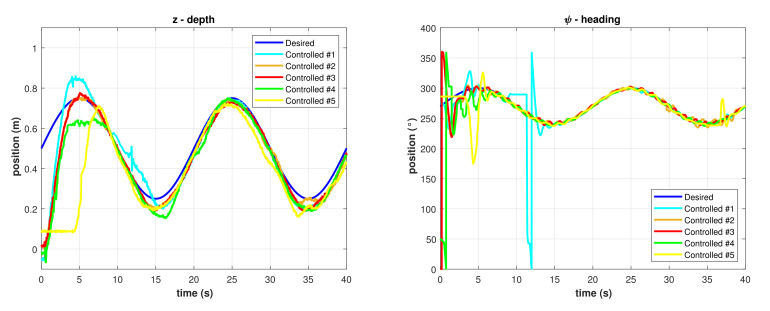
Controlled trajectories with the PID controller. (**Left**) Depth. (**Right**) Heading.

The functions in Equations (31) and (32) were programmed in the BlueROV2 as desired trajectories for depth and heading, respectively, meanwhile the desired roll was set to ϕ=0. The initial desired positions were set to $z_{i}=0.50$ m and $\psi_{i}=270$ °, the desired amplitudes were $A_{z}=0.25$ m and $A_{\psi }=30$ °, and the period was set to $T=\frac{1}{\omega }=20$ s. Results for different runs with the PID controller implementation are shown in Figure 7.

**Figure 8:**
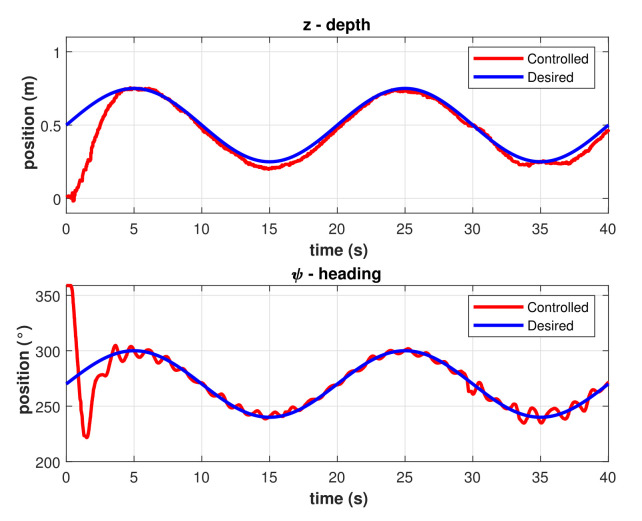
Controlled trajectories with the PID controller.

The best result for the PID controller is shown in Figure 8, a significant error can be observed in the depth trajectory control meanwhile the heading trajectory control error is significantly minor, but there is an overshoot in its transitory response.

**Figure 9:**
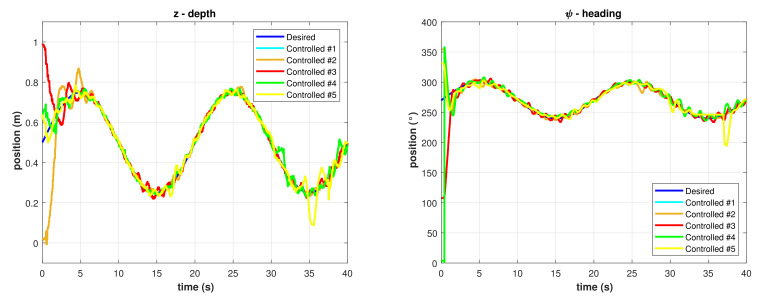
Controlled trajectories with the asymptotic model-free 2nd-order SMC. (**Left**) Depth. (**Right**) Heading.

**Figure 10:**
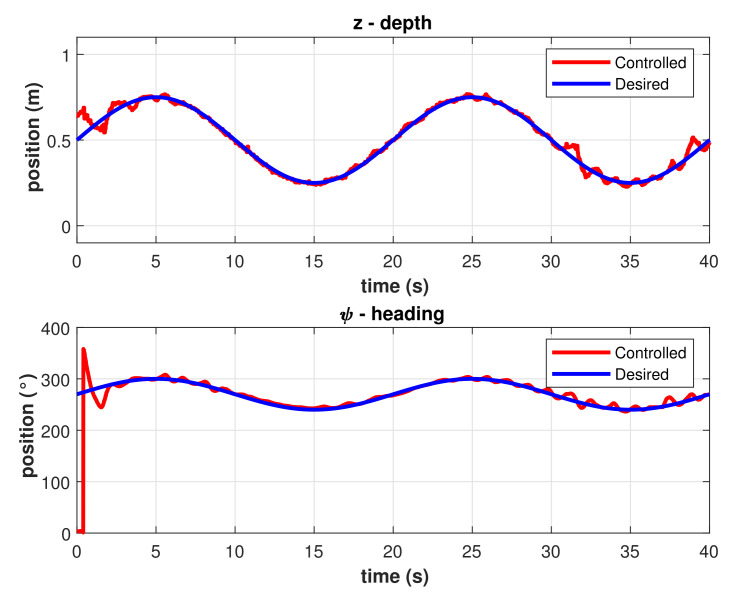
Best controlled trajectories with the asymptotic model-free 2nd-order SMC.

The same experiment was performed applying the model-free 2nd-order SMC with asymptotic convergence. Results for trajectory tracking of *z* and ψ in different runs of the experiment are shown in Figure 9. The graph in Figure 10 includes the best performance of the asymptotic 2nd-order SMC, there is a faster response compared with the PID controller, and the error once the robot meets the trajectory is smaller.

**Figure 11:**
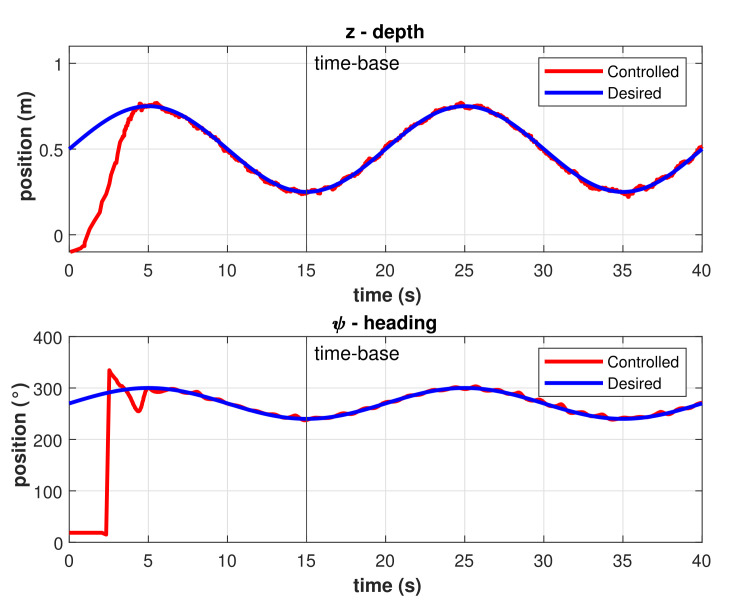
Controlled trajectory with the model-free high-order SMC with finite-time convergence in a 5 s predefined-time.

**Figure 12:**
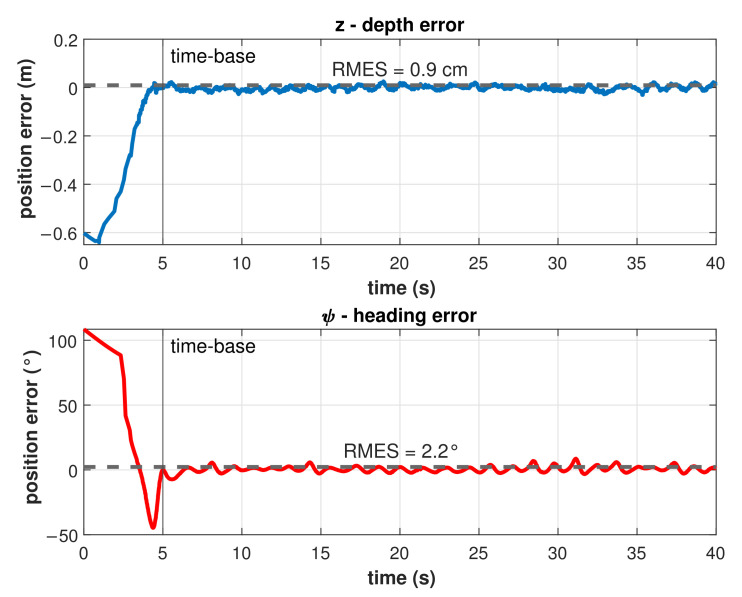
Tracking error for depth and heading control.

Results for the trajectory tracking by the model-free high-order SMC with finite-time convergence in a predefined time-base of 5 s are shown in Figure 11. Note that, at the beginning of the controlled trajectory, the BlueROV2 approaches slowly to the desired trajectory and then accelerates to reduce the error to practically zero in the selected time-base of 5 s. After the time-base is reached, the controller maintains the robot in the desired trajectory with a minimum Root Mean Square Error (RMSE) (<1 cm and <2.3°) as can be seen in Figure 12. Those errors can be attributed to hardware limitations such as sensor accuracy, resolution, and measurement rates, processor speed, communication baud rates, etc., which limits the control cycle frequency to a maximum of 10 Hz compared with a frequency 100 to 1000 times higher in simulations.

**Figure 13:**
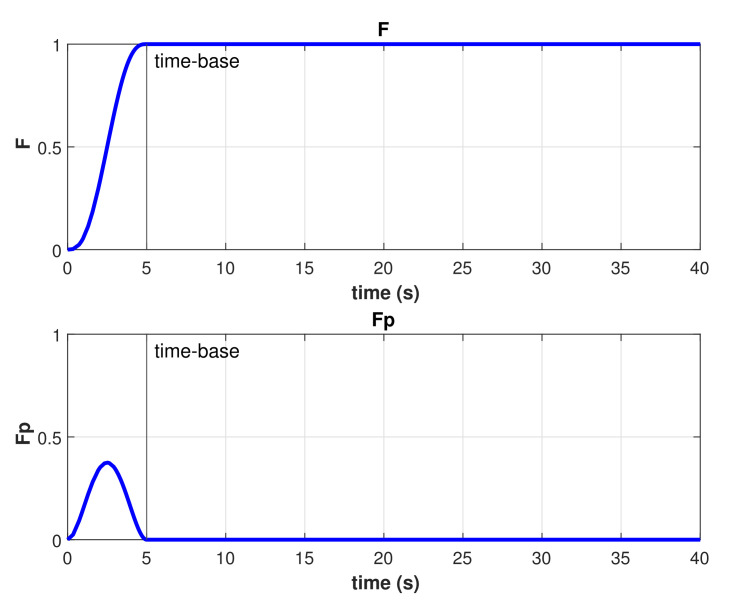
Time base generator results for $t_{b}=5$ s.

**Figure 14:**
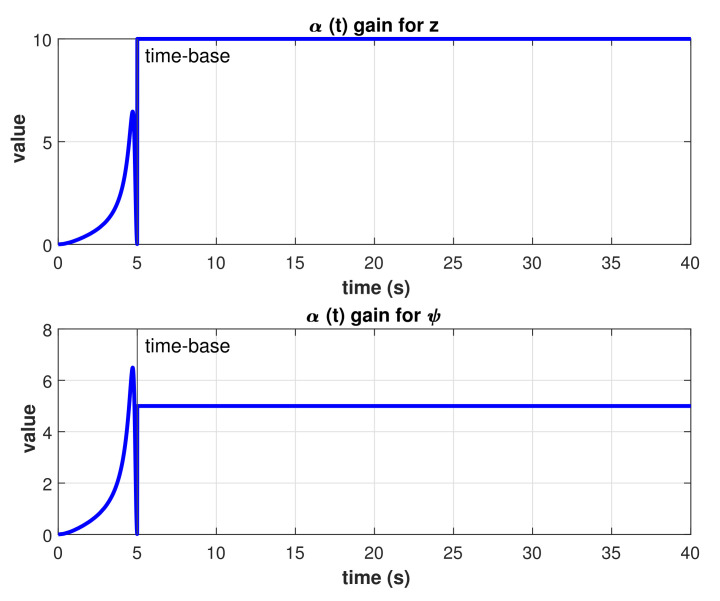
Time-varying gain α(t) result for $t_{b}=5$ s.

**Figure 15:**
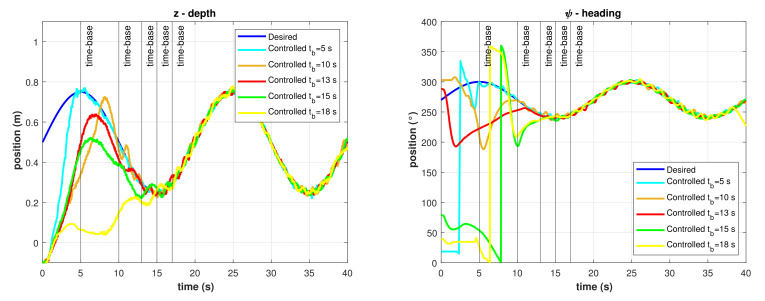
Controlled trajectories with the model-free high-order SMC with finite-time convergence in predefined-time. (**Left**) Depth. (**Right**) Heading.

The resulting TBG for this experiment is shown in Figure 13. As described in Equations (27) and (28), there is a smooth transition from zero to one which is completed exactly at the given time-base. The resulting time-varying gain α(t) is shown in Figure 14, as described in Equation (29). Several runs of the same experiment were performed with the model-free high-order SMC with finite-time convergence defining different time-bases. Results of the controlled depth and yaw trajectories are shown in Figure 15. It can be observed that the controller drives the robot to the desired trajectory to match it in the desired time-base for all cases.

**Figure 16:**
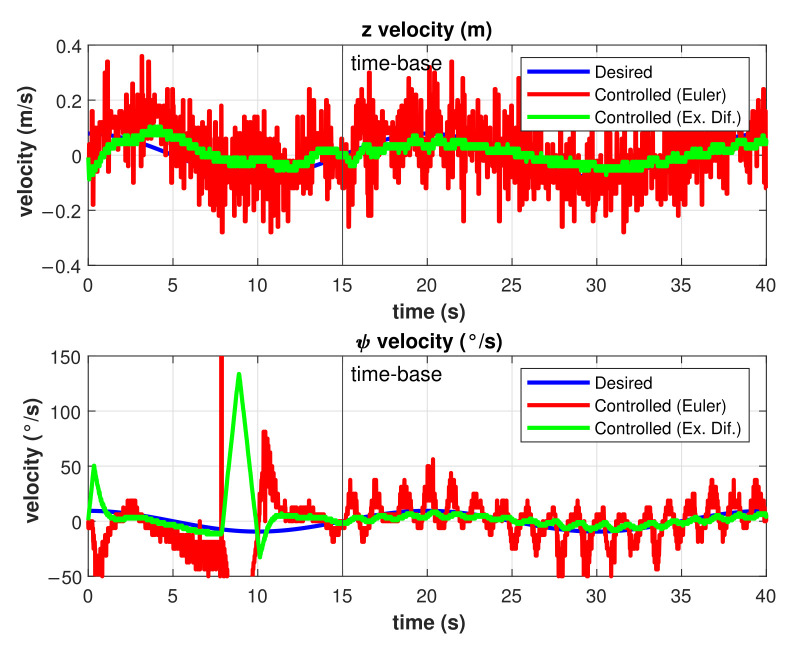
Velocities estimations by Euler and Exact differentiators.

Velocities estimations are another source of noise and, therefore, errors in the trajectory tracking problem for the SMC. To reduce these errors, the exact differentiator described in Equation (38) was used for the experiments in both the asymptotic and finite-time SMC. The results of the $\dot{z}$ and $\dot{\psi }$ estimations for a finite-time controlled trajectory are shown in Figure 16. As can be observed, the uncertainty in the estimations was reduced significantly by the use of the exact differentiator.

**Figure 17:**
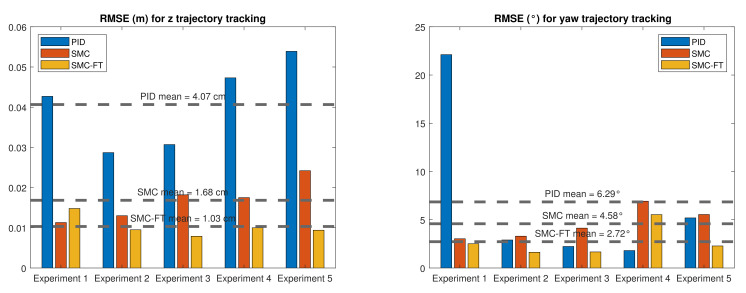
RMSE comparison for the trajectory tracking with the different controllers. (**Left**) Depth. (**Right**) Heading.

The RMSE was computed as a performance indicator of the different controllers applied. As the error varies during the convergence, the RMSE was calculated after the robot is in the desired trajectory. For the finite-time controller, the robot is considered in the trajectory when $t>t_{b}$. For the PID and the asymptotic SMC controllers, the robot is considered in trajectory once the controlled trajectory intersects with the desired trajectory. Results for the different control approaches are shown in Figure 17.

In the depth trajectory tracking, the mean RMSE—considering all the runs of the experiment—is 4.07 cm for the PID control (PID), 1.68 cm for the asymptotic 2nd-order SMC (SMC), and the best was the high-order SMC with finite-time convergence (SMC-FT) with a value of 1.03 cm. For the yaw trajectory tracking, the mean RMSE was 6.29° for the PID control, 4.58° for the asymptotic 2nd-order SMC, and, again, the best was the high-order SMC with finite-time convergence with a value of 2.72°.

**Figure 18:**
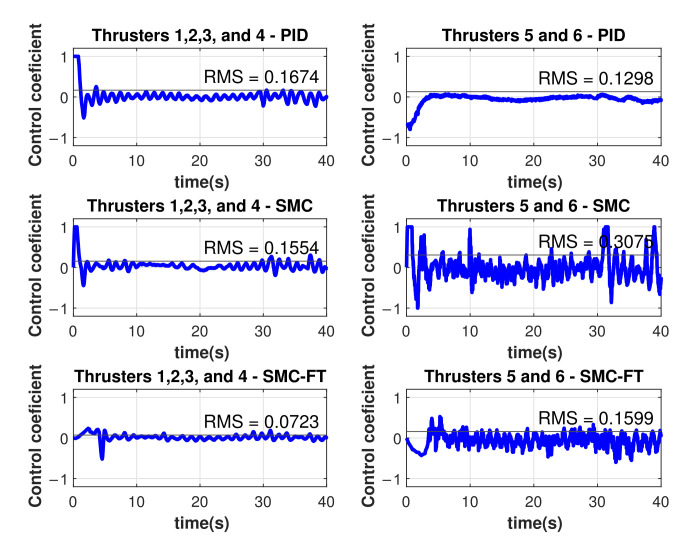
Control coefficients on the thrusters for the different control approaches.

Another performance indicator is the energy consumption in the thrusters. A control coefficient of +1.0 represents the maximum thrust in one direction and a control coefficient of −1.0 represents the maximum thrust in the opposite direction, in both cases, the power consumption is 625 W approximately. A higher magnitude of this coefficient means a bigger energy consumption from the corresponding thruster. The control coefficient for the different thrusters as result of the experiments shown in Figure 8, Figure 10 and Figure 11 are shown in Figure 18.

The resulting control coefficients for thrusters 1, 2, 3, and 4 were identical in magnitude, the same occurred for thrusters 5 and 6. The Root Mean Square (RMS) values of the control coefficients were computed as performance indicator as follows

(40) $$u_{rms}=\sqrt{\frac{1}{N}\sum_{i=1}^{N}u_{i}^{2}},$$

where *N* is the number of samples. Considering the six thrusters, the RMS value of the control coefficient was 0.155 for the PID controller, 0.206 for the asymptotic SMC, and 0.101 for the finite-time SMC with $t_{b}=5$ s. The finite-time controller was able to drive the vehicle to the desired trajectory with 35 % less energy demanded than the PID controller, even when they reach the desired trajectory at the same time. The asymptotic SMC was faster in its approach to the desired trajectory, resulting in an energy demand twice as bigger as the demanded by the finite-time controller.

As can be observed in the different performance indicators, the finite-time SMC proposed outperforms the asymptotic SMC even when, as stated in previous sections, they were programmed with the same parameters and gains—except for α(t)—which is a clear example of the advantages of using a finite-time convergence solution. Performing a tuning on the PID or asymptotic SMC to achieve a faster or slower convergence will also modify the performance of the controller in the trajectory tracking. This does not happen with the finite-time controller, where a simple change in the $t_{b}$ parameter will lead to a faster or slower convergence maintaining its performance and robustness. The user can benefit from this to obtain the best performance of the controller according to the needs demanded by the task.

## 5. Conclusions

In this work, a model-free high-order SMC with finite-time convergence in a predefined time was tested and validated through experimentation in a water pool, using the BlueROV2 robot. This robot was modified and instrumented to be able to follow time-parametrized trajectories at depth and orientation. Results showed that the finite-time convergence controller was able to drive the robot to the desired trajectory in a desired predefined time and maintain it there with a small error for all of the experiment runs. The performance of the controller was then compared with a PID controller and an asymptotic 2nd-order SMC in terms of the RMSE. The mean depth error after the total of the experiments was 75% smaller for the finite-time convergence controller compared with the PID control. Additionally, it was 38% smaller compared with the asymptotic 2nd-order SMC which kept the same parameters and gains of the finite-time controller with the exception of the α gain. Regarding the yaw trajectory tracking, the same indicator was 57% and 41% smaller for the finite-time controller when compared to the PID and asymptotic SMC, respectively. Although it was expected that the finite-time controller outruns the PID control performance, the fact that it also outperformed its asymptotic equivalent demonstrates the advantages of a finite-time convergence controller in the AUV navigation problem. Another advantage of the use of this finite-time controller is the smaller energy consumption. The RMS thruster coefficient was 35% smaller when compared with the PID solution with both controllers converging at the same time. It was also half of the RMS coefficient computed for the asymptotic SMC. Future work will expand the experimentation to *x* and *y* axes. Additionally, this controller will be implemented and tested for cooperative intervention missions of AUVs, where it is important that the vehicles can reach a desired position or trajectory at a predefined time. This controller will be especially useful considering scenarios where the communication between AUVs is limited or does not exist at all.

## Data Availability

Data sharing is not applicable to this article.

## Abbreviations

The following abbreviations are used in this manuscript:

AUV	Autonomous Underwater Vehicles
DoF	Degrees Of Freedom
ESC	Electronic Speed Controllers
PID	Proportional Integrate Derivative
PWM	Pulse Wide Modulation
RMS	Root Mean Square
RMSE	Root Mean Square Error
ROV	Remotely Operated Vehicle
ROS	Robot Operating System
RPi	Raspberry Pi
SMC	Sliding Mode Control
SNAME	Society of Naval Architects and Marine Engineers
TBG	Time-Base Generator
